# Anisocoria without extraocular muscle impairment due to moderate traumatic brain injury with midbrain contusion: a case report

**DOI:** 10.1186/s12883-023-03331-2

**Published:** 2023-07-15

**Authors:** Koshi Ota, Hitoshi Kobata, Yoshisuke Hamada, Saki Mizutani, Terunari Okuyama, Kanna Ota, Yuriko Takeda, Akira Takasu

**Affiliations:** Department of Emergency and Critical Care Medicine, Osaka Medical and Pharmaceutical University, 2-7 Daigaku-machi, Takatsuki City, 596-8686 Osaka Japan

**Keywords:** Anisocoria, Automated infrared pupillometry (AIP), Traumatic brain injury (TBI)

## Abstract

**Background:**

New-onset anisocoria is an important clinical clue to life-threatening intracranial injury. Anisocoria alone without impairment of extraocular muscles is a rare presentation of moderate traumatic brain injury (TBI).

**Case presentation:**

A 79-year-old woman was transported to hospital soon after falling off a bicycle. Glasgow Coma Scale score on arrival was 11 (E3V3M5). On examination at admission, she was found to be drowsy. Bruising was seen around the right eye and pupil diameters differed (right, 4.5 mm; left, 3.0 mm; both reactive to light). Computed tomography of the head revealed hemorrhagic contusion in the left temporal lobe and left pretectal area of the midbrain, right clavicular fracture, and pulmonary contusion with fractures of the 3rd and 4th ribs. Magnetic resonance imaging confirmed hemorrhagic contusion of the midbrain. The patient achieved full recovery of motor and mental functions with conservative treatment and was discharged on hospital day 17.

**Conclusion:**

We encountered a case of anisocoria without major extraocular muscle impairment due to moderate TBI with midbrain contusion.

**Supplementary Information:**

The online version contains supplementary material available at 10.1186/s12883-023-03331-2.

## Background

The presence of a large, unreactive pupil in the form of anisocoria (new-onset pupil size difference of ≥1 mm) is an important clinical clue to life-threatening intracranial injury [1, 2]. Anisocoria and lower baseline Glasgow Coma Scale (GCS) score are reportedly associated with moderate disability and poor recovery from traumatic brain injury (TBI) [3].

Anisocoria is generally caused by either impaired constriction (parasympathetic pathway) or impaired dilation (sympathetic pathway) of the pupils [4]. Injury in either pathway can result in changes to pupil size. Compressive lesions resulting from head trauma typically involve the pupil via the superficial parasympathetic fibers of the oculomotor nerve, which innervates the pupil. Oculomotor nerve palsy rarely presents as isolated mydriasis; associated findings include ptosis, an ipsilateral down-and-out position, and loss of accommodation.

Including several variations, the prevalence of TBI is reportedly around 800–1100 cases per 100,000 population in Southeast Asia region [5]. 75% of fatalities occur among men, with patients ≥65 years old accounting for the greatest proportion of fatal injuries [6]. The leading external causes of fatal TBI were firearm-related (39% of reported fatalities), traffic accidents (34%), and fall-related (10%).^6^ According to the Japan Neurotrauma Data Bank (JNTDB) project 2015, the prevalence of patients > 65 years old with severe TBI was 51.7%, and the most common mechanisms of injury in Japan for geriatric TBI patients were falls (54.8%) and traffic accidents (33.7%) [7].

With the ongoing aging of society, emergency physicians are likely to treat more geriatric TBI patients, with or without anisocoria.

Here, we describe the case of an elderly Japanese woman with anisocoria without impairment of the extraocular muscles due to moderate TBI with midbrain hemorrhage.

## Case presentation

A 79-year-old Japanese woman was transported to our hospital soon after falling off a bicycle. She had fallen after running into a young man riding another bicycle. The young man had called an ambulance after she appeared unresponsive to stimuli and she was transferred to our hospital. The patient had a medical history of gastroesophageal reflux disease and hypoacusis requiring hearing aids, and had been prescribed esomeprazole at 10 mg once daily and mosapride citrate hydrate at 5 mg three times daily. Her habitual activities and familial history were unremarkable. She was a part-time cleaner and lived with her husband. On arrival at the emergency room, vital signs were: temperature, 35.8 °C; heart rate, 58 beats/min with regular rhythm; respiratory rate, 20 breaths/min; blood pressure, 150/79 mmHg; and oxygen saturation, 100% with supplemental oxygen at 10 L/min via simple oxygen mask. Glasgow Coma Scale score on arrival was 11 (E3V3M5), indicating affected consciousness due to brain injury. On examination, the patient was drowsy but responsive to pain stimuli. The trachea was central, and neither crackles nor decreased breath sounds were heard on auscultation. The abdomen was not distended. Examination of cranial nerves was difficult due to drowsiness. Bruising was seen around the right eye. Pupil diameters differed, at 4.5 mm on the right and 3.0 mm on the left. Both eyes showed reactivity to light. Automated infrared pupillometry (AIP) and neurological pupil index (NPi) were applied as objective means of assessing the pupillary light reflex (PLR) using an NPi-200 pupillometer (NeurOptics, Inc., Irvine, CA, USA). It showed anisocoria with dilated right pupil (Video).

Examination of the limbs showed no abnormalities other than bruising over the right clavicle.

**Fig. 1:**
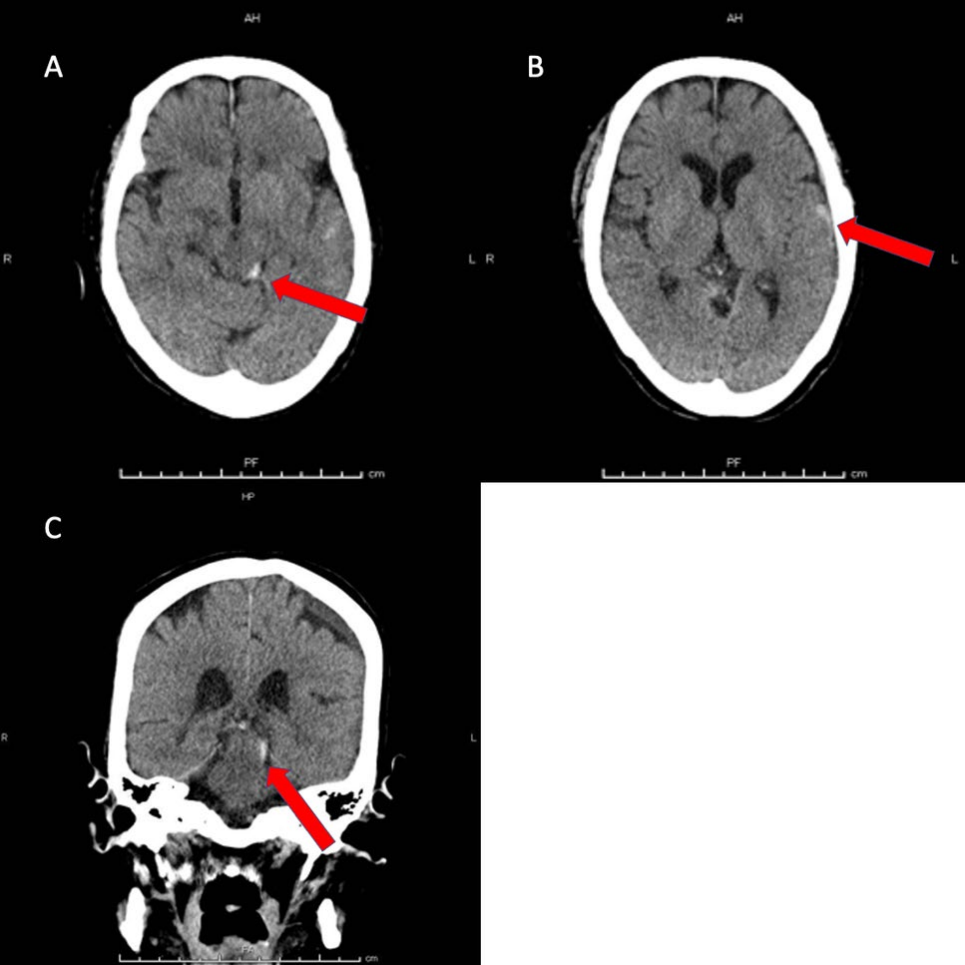
Axial computed tomography (CT) shows subarachnoid hemorrhage (SAH) around the midbrain (**A**) and left temporal lobe (**B**). Coronal CT of the midbrain shows SAH and brain contusion (**C**). Red arrows show the respective lesions

**Fig. 2:**
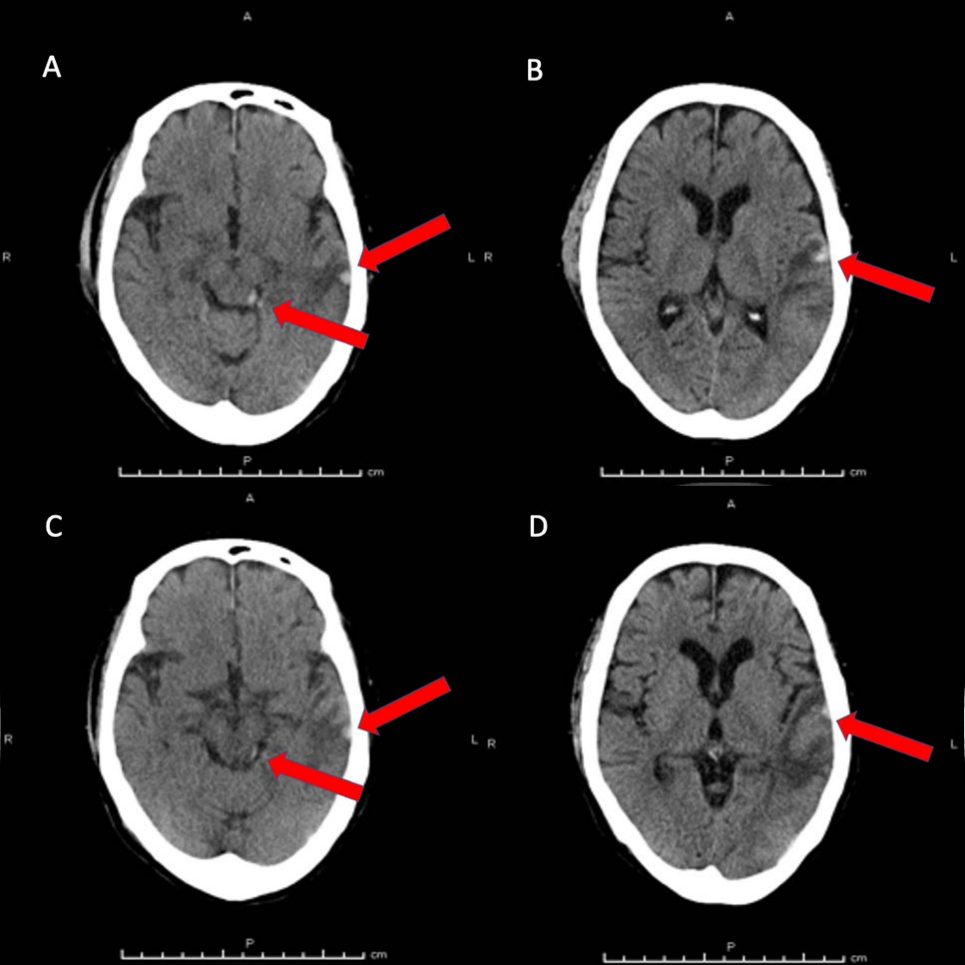
Axial CT on hospital day 2 (**A** and **B**) and on hospital day 7 (**C** and **D**). No enlargement of hemorrhages is evident. Red arrows show the respective lesions

Computed tomography (CT) of the head revealed hemorrhagic contusion in the left temporal lobe and left pretectal area of the midbrain, shaped by the cerebellar tentorium (Fig. 1A-C). Right clavicular fracture and pulmonary contusion with fractures of the 3rd and 4th ribs were also seen. CT the following day and on hospital day 5 showed no enlargement of hemorrhage (Fig. 2A-D). Cranial examinations showed normal results other than anisocoria (right pupil, 4.5 mm; left pupil, 3.0 mm; reactive to light) the following day. Ocular motility was normal (Supplemental Fig. 1).

On observation with an infrared pupillometer (NPi-200; NeurOptics, Inc.), the NPi was 3.2 on the right and 4.4 on the left, with pupil diameters of 5.66 mm and 3.57 mm, respectively. Abnormal pupillary findings persisted, with a difference in NPi of 2.0 on hospital day 5 and 1.5 on hospital day 7 (Table 1). Open reduction and plate fixation were performed to treat the right clavicular fracture on hospital day 7.

**Table 1:** Automated infrared pupillometry (AIP) on hospital day 5 and 7

Hospital day 5			
	Right	Left	Differential
NPi	3.2	4.4	L > R 1.2
Size (mm)	5.66	3.57	R > L 2.09
MIN (mm)	4.10	2.50	R > L 1.60
CH (%)	28	30	
CV (mm/s)	2.22	2.18	
MCV (mm/s)	4.11	3.77	
LAT (s)	0.23	0.20	
DV (mm/s)	0.98	1.35	
Hospital day 7			
	Right	Left	Differential
NPi	2.0	3.5	L > R 1.5
Size (mm)	6.79	4.56	R > L 2.23
MIN (mm)	5.69	3.33	R > L 2.36
CH (%)	16	27	
CV (mm/s)	2.16	3.07	
MCV (mm/s)	3.06	4	
LAT (s)	0.23	0.17	
DV (mm/s)	0.81	1.26	

*NPi*  Neurological pupil index, *Size*  Maximum diameter: maximum pupil size before constriction, *MIN*  Cinimum diameter: pupil diameter at peak constriction, *CH*  Change: (Size - MIN)/Size as a percentage, *MCV*  Maximum constriction velocity: maximum velocity of pupil constriction of the pupil diameter responding to a flash of light, measured in millimeters per second, *LAT*  Latency of constriction: time of onset of constriction following initiation of light stimulus

**Fig. 3:**
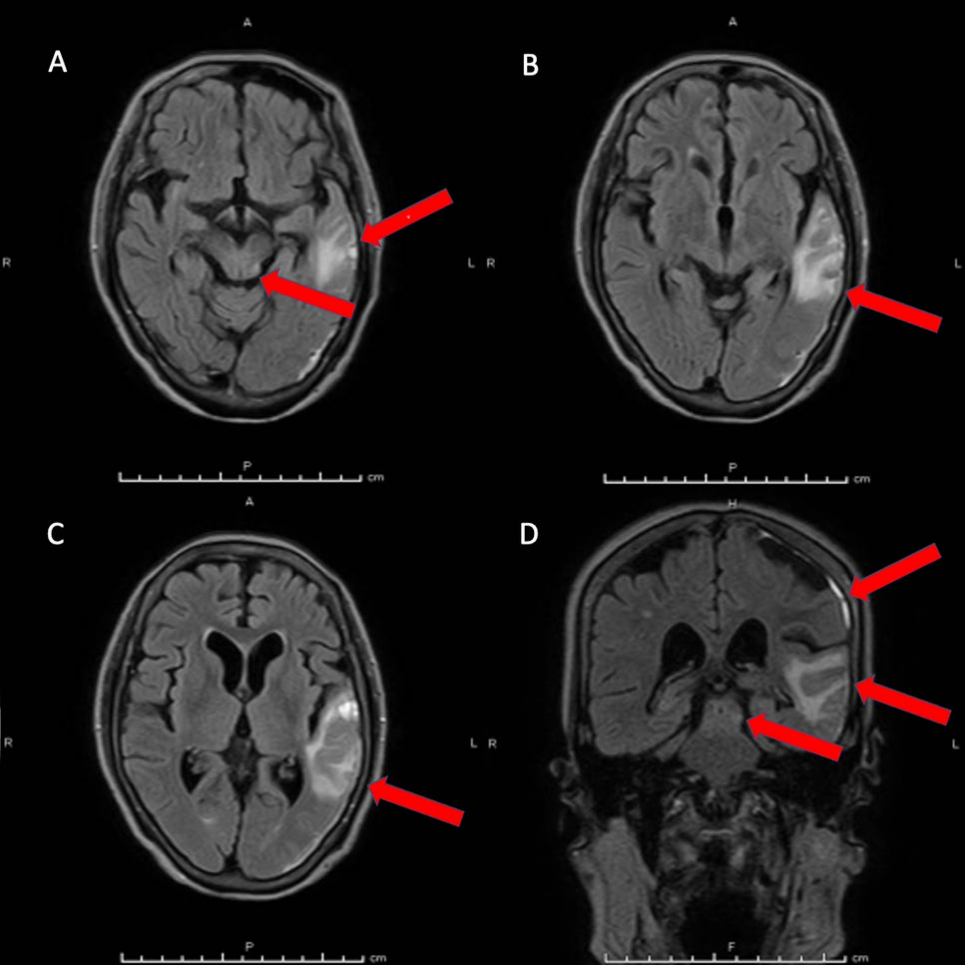
Magnetic resonance imaging (MRI) findings on hospital day 13. Signal hyperintensity on fluid-attenuated inversion recovery (FLAIR) is apparent in the midbrain (**A**) and left temporal lobe (**B**, **C**). Brain contusion is also seen in the left temporal lobe. Coronal-view FLAIR shows contusion in the midbrain and left temporal lobe and subdural hemorrhage in the left temporal lobe (**D**). Red arrows show the respective lesions

The postoperative course was uneventful and magnetic resonance imaging (MRI) of the head was performed on hospital day 13. MRI confirmed contusion in the left temporal lobe and left midbrain (Fig. 3A-D). The patient was discharged on hospital day 17 and left hospital able to walk unaided. Follow-up visits showed anisocoria remaining at 4 days and 32 days after discharge and she complained of dizziness and weight loss, but she was able to resume her normal lifestyle without visual disturbance. The NPi of the right eye recovered to 3.8 and pupillary size returned symmetry between both pupils as of the last follow-up visit (Supplemental Fig. 2).

## Discussion

We encountered a case of anisocoria alone without major impairment of extraocular muscles due to moderate TBI with contusion in the left pretectal area of the midbrain. Anisocoria represents an important clinical clue to intracranial injury, suggesting uncal herniation. The patient in this case was able to undergo CT directly after a brief examination.

A direct focal impact on the midbrain at the free edge of the tentorium could produce contusional hemorrhage in the midbrain, as seen in our case [7]. Typically, the location of this type of lesion is reported ipsilateral to the site of impact [7]. In our case, both contusions identified were attributable to the contrecoup mechanism. At the time of injury, the lateral midbrain surface collided with the more rigid structure of the tentorium, causing contusion in the midbrain.

Generally, when the midbrain is injured, the ipsilateral extra- and intraocular muscles are affected. The present case was unusual because only the contralateral intraocular muscles were involved. Several mechanisms could potentially result in anisocoria without major impairment of extraocular muscles. First, right eye contusion might have occurred in isolation, as direct trauma to the ciliary ganglion could damage the parasympathetic system, as could traction on the inferior oblique muscle and its nerve supply. However, the persistence of right eye mydriasis on follow-up did not fit the model of simple right eye contusion and the fact that extraocular muscles were almost normal likewise did not fit right oculomotor nerve palsy. Second, the injury might have impacted the right side of the midbrain, where the oculomotor nucleus is located. This theory is also unlikely, since only the Edinger-Westphal nucleus, which controls the parasympathetic nervous system, should be selectively damaged. Furthermore, neither CT nor MRI showed right midbrain lesions. Third, the accident could have caused compression of the nerve between the posterior cerebral artery and superior cerebellar artery arising from caudal displacement of the right brainstem, but we could not confirm evidence of such compression. Fourth, the patient might have already had anisocoria before the accident, since physiological anisocoria is reportedly found in about 20% of the normal population [8]. However, she did not mention physiological anisocoria on follow-up visits. Further, the NPi of the right eye was 2.0 on hospital day 5 and recovered to 3.8 as of the last follow-up visit and pupillary size returned symmetry between both pupils at 10 months after discharge, which would seem to reduce the likelihood of physiological anisocoria [9]. Although the mechanisms underlying pupillary anomalies in this case remain uncertain, strong shock to the midbrain may have been involved. Our patient recovered the NPi faster than pupillary size. Early improvements in NPi scores in our patient correlated with a better prognosis for the return of pupillary function but anisocoria remained for 10 months. Even though the NPi was 1.5 on the right on hospital day 7, initial NPi was 3.2 on the right indicated that complete return of pupillary function between both pupils could be expected.

The incidence of TBI has increased in elderly adults in Japan with the aging of society [10]. Among individuals ≥75 years old, pupil abnormalities and traumatic subarachnoid hemorrhage were reportedly associated with an increased probability of unfavorable outcomes at 6 months after injury in a nationwide patient cohort in Japan [11]. The presence of a difference in the NPi between pupils was another prognostic factor for poor neurological outcomes [9]. Fortunately, our patient achieved full recovery of motor and mental functions with conservative treatment, even though we could not elucidate the reasons for anisocoria and good neurological outcome.

## Conclusion

Anisocoria is an important clinical clue to life-threatening intracranial injury and rarely presents as isolated mydriasis. We encountered a patient presenting with anisocoria alone without major impairment of extraocular muscles. Midbrain injury was potentially associated with this uncommon presentation.

## Supplementary Information

**Additional file 1. **Ocular motility exam shows normal results in 5positions. No abnormalities were found.

**Additional file 2.**

**Additional file 3.**

## Data Availability

The datasets used and/or analysed during the current study available from the corresponding author on reasonable request.

## Abbreviations

AIP: Automated infrared pupillometry
*CT*: Computed tomography
*NPi*: Neurological pupil index
*MRI*: Magnetic resonance imaging
TBI: Traumatic brain injury
