# Supplementary figures and images for: Anisocoria without extraocular muscle impairment due to moderate traumatic brain injury with midbrain contusion: a case report
Source: BMC Neurol. 2023 Jul 15;23:270. doi: 10.1186/s12883-023-03331-2 (PMC10349450; doi:10.1186/s12883-023-03331-2)

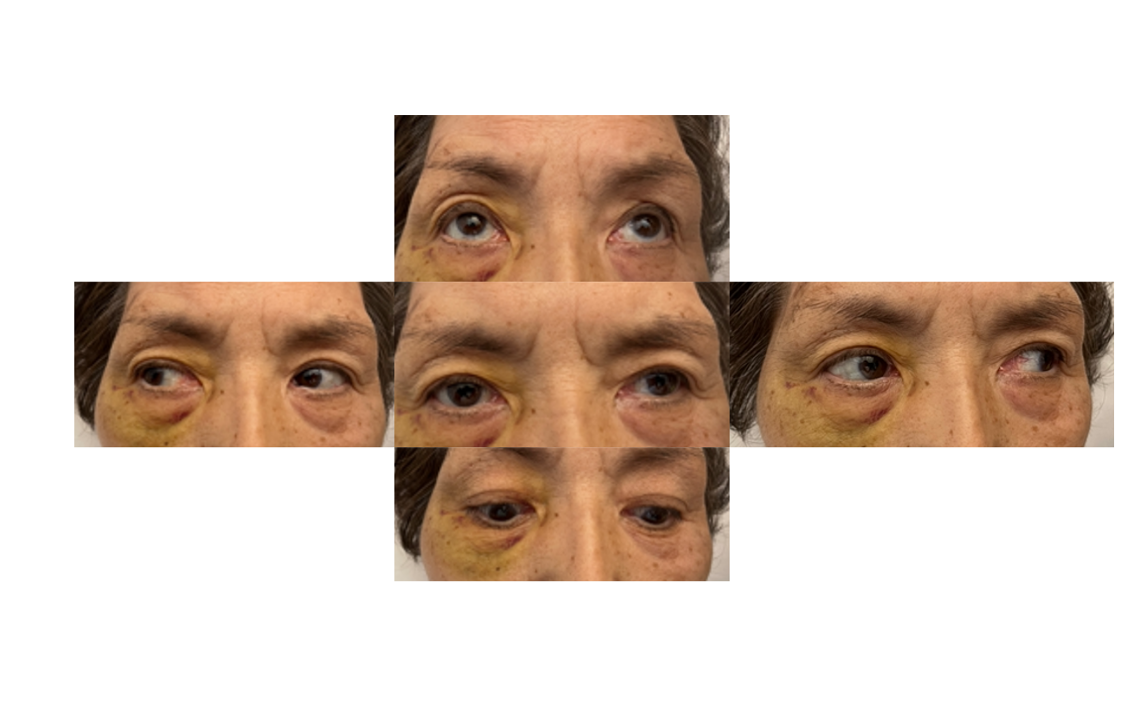

Supplement: Supplementary file 1 — Additional file 1. Ocular motility exam shows normal results in 5positions. No abnormalities were found. [file 12883_2023_3331_MOESM1_ESM.tiff]

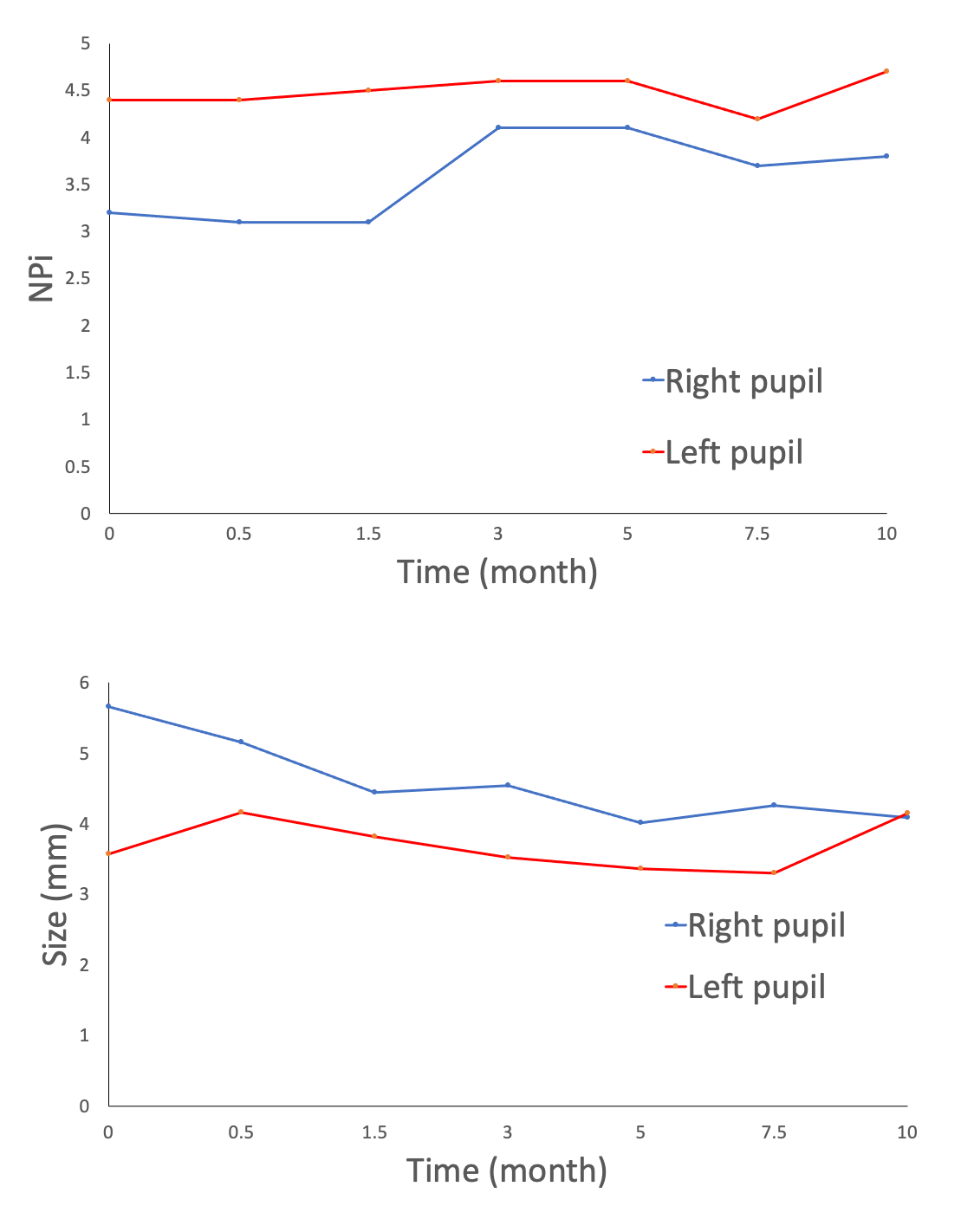

Supplement: Supplementary file 3 — Additional file 3. [file 12883_2023_3331_MOESM3_ESM.tiff]
